# Does inferior vena cava respiratory variability predict fluid responsiveness in spontaneously breathing patients?

**DOI:** 10.1186/s13054-015-1100-9

**Published:** 2015-11-13

**Authors:** Norair Airapetian, Julien Maizel, Ola Alyamani, Yazine Mahjoub, Emmanuel Lorne, Melanie Levrard, Nacim Ammenouche, Aziz Seydi, François Tinturier, Eric Lobjoie, Hervé Dupont, Michel Slama

**Affiliations:** Intensive Care Unit, Department of Nephrology, Amiens University Medical Center, 80054 Cedex 1, Amiens, France; Department of Anesthesiology and Intensive Care, Amiens University Medical Center, Amiens, France; INSERM U-1088, Jules Verne University of Picardie, Amiens, France

## Abstract

**Introduction:**

We have almost no information concerning the value of inferior vena cava (IVC) respiratory variations in spontaneously breathing ICU patients (SBP) to predict fluid responsiveness.

**Methods:**

SBP with clinical fluid need were included prospectively in the study. Echocardiography and Doppler ultrasound were used to record the aortic velocity-time integral (VTI), stroke volume (SV), cardiac output (CO) and IVC collapsibility index (cIVC) ((maximum diameter (IVCmax)– minimum diameter (IVCmin))/ IVCmax) at baseline, after a passive leg-raising maneuver (PLR) and after 500 ml of saline infusion.

**Results:**

Fifty-nine patients (30 males and 29 females; 57 ± 18 years-old) were included in the study. Of these, 29 (49 %) were considered to be responders (≥10 % increase in CO after fluid infusion). There were no significant differences between responders and nonresponders at baseline, except for a higher aortic VTI in nonresponders (16 cm vs. 19 cm, p = 0.03). Responders had a lower baseline IVCmin than nonresponders (11 ± 5 mm vs. 14 ± 5 mm, p = 0.04) and more marked IVC variations (cIVC: 35 ± 16 vs. 27 ± 10 %, p = 0.04). Prediction of fluid-responsiveness using cIVC and IVCmax was low (area under the curve for cIVC at baseline 0.62 ± 0.07; 95 %, CI 0.49-0.74 and for IVCmax at baseline 0.62 ± 0.07; 95 % CI 0.49-0.75). In contrast, IVC respiratory variations >42 % in SBP demonstrated a high specificity (97 %) and a positive predictive value (90 %) to predict an increase in CO after fluid infusion.

**Conclusions:**

In SBP with suspected hypovolemia, vena cava size and respiratory variability do not predict fluid responsiveness. In contrast, a cIVC >42 % may predict an increase in CO after fluid infusion.

## Introduction

Hypovolemia is a very frequent clinical situation in the intensive care unit (ICU) and is primarily treated with volume expansion. Unfortunately, only 40–70 % of critically ill patients with acute circulatory failure display a significant increase in their cardiac output (CO) in response to volume expansion [1]. In septic shock, fluid infusion is usually recommended [2] but may be harmful - particularly in patients with acute respiratory distress syndrome [3, 4]. It is therefore essential to have reliable tools for predicting the efficacy of volume expansion and thus distinguishing patients who might benefit from volume expansion from those in whom the treatment is likely to be inefficacious. Many studies have focused on the prediction of fluid responsiveness, and many different dynamic markers of fluid responsiveness have been studied in recent years [1, 5–7]. Recent research has also demonstrated that standard static hemodynamic measurements (such as central venous pressure or pulmonary artery occlusion pressure) are of little value in predicting fluid responsiveness [1, 8]. Research has also demonstrated that inferior vena cava (IVC) diameter may predict central venous pressure in intubated, mechanically ventilated patients and in spontaneously breathing patients [9–11]. Inferior vena cava respiratory variability is known to be related to fluid-responsiveness in ICU mechanically ventilated patients and may discriminate between responder patients (i.e., in whom CO increases after fluid infusion) from nonresponders (in whom CO remains at the same level or increases only slightly) [12, 13]. However, data on the accuracy of IVC variations for predicting fluid needs in spontaneously breathing patients are scarce [14]. The aim of the present study was to determine the value of IVC respiratory variability in spontaneously breathing patients for predicting fluid responsiveness (rather than to analyze the relationship between IVC diameter and central venous pressure (CVP)).

## Methods

### Patients

This prospective study was performed in two ICUs at Amiens University Medical Center (Amiens, France). Nonintubated, nonventilated, spontaneously breathing patients in whom the attending physician decided to perform fluid expansion were consecutively included. The decision by the attending physician to perform fluid expansion in our unit is usually based on the following criteria: clinical signs of acute circulatory failure (systolic blood pressure < 90 mmHg, urine output < 0.5 ml/kg, tachycardia, mottled skin) and/or oligoanuria (diuresis below 20 ml/h or 0.5 ml/kg/h) and/or acute kidney failure; and/or clinical and laboratory signs of extracellular dehydration. The exclusion criteria were as follows: clinical signs of hemorrhage, inability to defer fluid challenge for several minutes, arrhythmia, use of compression stockings and a contraindication to passive leg raising (PLR). The study's objectives and procedures were approved by the local investigational review board (comité de protection des personnes Nord Ouest II; Amiens, France). No consent was needed for this observational and non-interventional study.

### Study design

All patients were placed in a semirecumbent position for baseline measurements. The SBP, diastolic blood pressure (DBP), pulse pressure (PP) and MBP were measured with an invasive arterial pressure monitoring system (Agilent Component Monitoring System, model M1205A (Agilent, Boeblingen, Germany) and the heart rate (HR) was recorded. Cardiac output and IVC diameters were measured using echocardiography. The bed was then automatically moved to induce PLR of 30° [15]; blood pressure values, HR, IVC diameters and CO were measured two minutes later. The patient was then returned to the initial position and 500 cc of saline solution were administered intravenously over 15 minutes. Blood pressure, HR, and echocardiographic measurements were then repeated.

### Measurements

The following clinical characteristics were recorded: age, gender, Simplified Acute Physiology Score II (SAPS II), weight, McCabe score, clinical problems, primary diagnosis, medical history (hypertension, diabetes mellitus, cardiomyopathy, chronic obstructive pulmonary disease or pulmonary embolism). Echocardiography was performed using the HP Sonos 2000 and Philips Envisor (Philips Medical Systems, Suresnes, France). In a parasternal two-dimensional (2D) view, the aortic diameter (AoD) was measured at the aortic valve insertion (aortic annulus). The aortic area (AA) was calculated as follows: AA = (π x AoD^2^) / 4. In an apical five-chamber view, aortic blood flow was recorded using pulsed Doppler, with the sampling volume located at the aortic annulus. The velocity-time integral (VTI) for aortic blood flow was calculated. Stroke volume (SV) and CO were calculated as follows: SV = VTI x AA and CO = SV x HR. Aortic area was considered to be stable throughout the experiment and so was measured at baseline only. It was used to calculate CO during PLR and after fluid infusion. The reported aortic VTI was the average of three to five consecutive measurements over a single respiratory cycle.

The IVC was examined subcostally in a longitudinal section. The IVC diameter was measured in M-mode coupled to 2D mode 2 cm before the IVC joined the right atrium. The M-mode tracing was perpendicular to the IVC. The IVC collapsibility index (cIVC, which reflects the decrease in the diameter upon inspiration) was calculated as (maximum diameter on expiration (IVCmax) – minimum diameter on inspiration (IVCmin))/ IVCmax. The intra- and inter-observer variabilities in the measurement of IVC diameter were 8.4 ± 6.6 % and 5.7 ± 2.7 %, respectively. Intra-observer reproducibility was 4.9 ± 4.3 % and 4.9 ± 4.3 % for CO and VTI, respectively, and interobserver reproducibility was 4.2 ± 3.4 % and 4.2 ± 3.4 %, respectively. All measurements were performed by echocardiography-trained intensivists.

### Statistical analysis

All continuous variables are expressed as the mean ± standard deviation (SD). The Kolmogorov-Smirnov test was used to check the normality of data distribution. Relationships between variables were analyzed by linear regression. Intergroup comparisons of continuous and categorical variables were performed with Student's T test and the chi-squared test, respectively.

The patients were classified as responders (in whom CO increased by ≥ 10 % after fluid expansion compared with baseline) and nonresponders (in whom CO increased by < 10 %). Absolute values at baseline and changes in HR, pressure values, VTI (ΔVTI), SV (ΔSV), CO (ΔCO) and IVC diameter during PLR were analyzed. The correlation between these variables and changes in CO after fluid expansion and their value for predicting an increase in CO after fluid expansion were calculated by plotting a receiver operating characteristic (ROC) curve. The area under the curve (AUC) was calculated for all parameters and compared in a Hanley-McNeil test. Sensitivity, specificity, negative and positive predictive values, negative and positive likelihood ratios and the percentage of correct classification were calculated after defining a cut-off value. The threshold for statistical significance was set to p < 0.05. Statistical analysis was performed with MedCalc software (version 12.2.1, MedCalc Software, Mariakerke, Belgium).

## Results

The characteristics of the study population are summarized in Table 1. Fifty-nine patients (30 men and 29 women; mean age: 57 ± 18) were included in the study. They were variously suffering from hypotension or acute circulatory failure (n = 16; 27 %), oligoanuria or acute kidney failure (n = 20; 34 %) or clinical and laboratory signs of dehydration (n = 23; 39 %). Thirty-nine patients (58 %) were admitted to the ICU for non-surgical reasons and 25 patients (42 %) were admitted for surgical reasons. Only two patients were taking vasoactive agents at the time of inclusion.

**Table 1:** Characteristics of the study population

	Total study population (*n* = 59)	Responders (*n* = 29)	Nonresponders (*n* = 30)	p
Age (years), mean ± SD	57 ± 18	60 ± 17	54 ± 18	0.23
Male, n (%)	30 (51)	15(62)	15(50)	0.09
McCabe group 0/1/2, n	36/21/2	18/10/1	18/11/1	0.93
SAPS II, mean ± SD	30 ± 16	34 ± 17	26 ± 14	0.06
Non-surgical/surgical admissions n (%)	34/25(58/42)	17/12(62/38)	17/13(53/47)	0.49
Medical history, n (%):				
Hypertension	23(39)	11(38)	12(40)	0.71
Diabetes mellitus	10(17)	5(17)	5(16)	0.45
Cardiomyopathy	10(17)	4(14)	6(20)	0.68
- ischemic	7(12)	3(10)	4(14)	0.65
- hypertensive	2(3)	1(4)	1(3)	0.53
- obstructive	1(2)	0(0)	1(3)	1
Supraventricular arrhythmia	2(3)	1(3)	1(3)	0.47
COPD	9(15)	4(14)	5(16)	0.41
Pulmonary embolism	1(2)	0(0)	1(3)	1

*SAPS II* Simplified Acute Physiology Score II, *COPD* chronic obstructive pulmonary disease

Twenty-nine patients (49 %) were considered to be responders, with an increase in CO of 10 % or more after fluid challenge. There were no significant differences between responders and nonresponders in terms of demographic and baseline clinical characteristics (Table 1).

The variations in hemodynamic parameters and echocardiographic indices at baseline, during PLR, and after the fluid challenge are shown in Table 2. There were no significant differences between responders and nonresponders at baseline, with the exception of higher aortic VTI in nonresponders (16 cm vs. 19 cm, respectively; p = 0.03). After fluid infusion and after PLR, VTI, SV and (by definition) CO increased in responders but remained stable in nonresponders.

**Table 2:** Variations in hemodynamic parameters at baseline, after PLR and after fluid challenge in responders and nonresponders

	Responders (*n* = 29)	Nonresponders (*n* = 30)	p
SBP, mmHg			
Baseline	123 ± 28	117 ± 23	0.06
PLR	125 ± 26	123 ± 23	0.77
Volume expansion	126 ± 20	123 ± 25	0.61
DBP, mmHg			
Base	67 ± 17	72 ± 43	0.60
PLR	69 ± 15	68 ± 21	0.79
Volume expansion	67 ± 13	68 ± 21	0.82
MBP, mmHg			
Baseline	86 ± 19	87 ± 33	0.87
PLR	88 ± 18	86 ± 19	0.96
Volume expansion	87 ± 14	87 ± 21	0.95
HR, bpm			
Baseline	98 ± 16	96 ± 20	0.66
PLR	95 ± 16*	94 ± 20	0.72
Volume expansion	95 ± 15*	92 ± 20	0.54
VTI, cm			
Baseline	16 ± 4	19 ± 4	0.03
PLR	18 ± 4*	18 ± 4	0.90
Volume expansion	20 ± 4*†	18 ± 4	0.04
SV, ml			
Baseline	48 ± 13	53 ± 15	0.14
PLR	54 ± 12*	52 ± 15	0.68
Volume expansion	59 ± 12*†	52 ± 15	0.04
CO, L/min			
Baseline	4.6 ± 1.1	5.0 ± 1.5	0.21
PLR	5.1 ± 1.3*	4.8 ± 1.5	0.44
Volume expansion	5.6 ± 1.2*†	4.7 ± 1.6	0.02

Values are expressed as the mean ± SD

*PLR* passive leg-raising, *SBP* systolic blood pressure, *DBP* diastolic blood pressure, *MBP* mean blood pressure, *PP* pulse pressure, *HR* heart rate, *VTI* aortic velocity-time integral *SV* stroke volume, *CO* cardiac output

* = *p* < 0.05 vs. baseline

† = *p* < 0.05 vs. PLR

The responders' and nonresponders' echocardiographic parameters for the IVC at baseline and after fluid challenge are shown in Table 3. At baseline, responders had a smaller IVCmin than nonresponders (11 ± 5 vs. 14 ± 5 mm, respectively; p = 0.04) and displayed more marked IVC variations (cIVC 35 ± 16 vs. 27 ± 10 %, p = 0.04). After volume expansion, the two groups of patients differed significantly in terms of all IVC parameters, with again a greater cIVC in responders (35 ± 16 % at baseline and 18 ± 10 % after fluid challenge).

**Table 3:** Variations in echocardiographic parameters for the IVC at baseline, after PLR and after fluid challenge in responders and nonresponders

	Responders (*n* = 29)	Nonresponders (*n* = 30)	p
IVCmin, mm			
Baseline	11 ± 5	14 ± 5	0.04
PLR	16 ± 4*	15 ± 6	0.52
Volume expansion	12 ± 5	16 ± 5*	0.004
IVCmax, mm			
Baseline	17 ± 4	19 ± 4	0.07
PLR	19 ± 4	19 ± 5	0.90
Volume expansion	16 ± 4	19 ± 5	0.01
cIVC, %			
Baseline	35 ± 16	27 ± 10	0.04
PLR	19 ± 10*	28 ± 18*	0.02
Volume expansion	18 ± 10*	28 ± 18*	0.02

Values are expressed as the mean ± SD

*IVCmax* maximum inferior vena cava diameter, *PLR* passive leg raising, *IVCmin* minimum inferior vena cava diameter, *cIVC* inferior vena cava collapsibility index

* = *p* < 0.05 vs. baseline

**Fig. 1:**
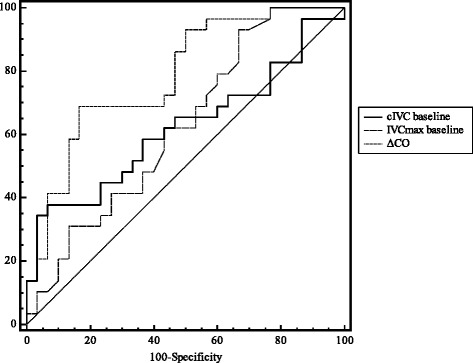
Receiver operating characteristic curves for discriminating between volume expansion responders and nonresponders. *∆CO* change in CO between baseline and after PLR, *VCmax* maximum inferior vena cava diameter at baseline, *cIVC* inferior vena cava collapsibility index at baseline, *PLR* passive leg raising

The only significant correlation was between changes in CO during PLR challenge and changes in CO after volume expansion (r = 0.69, p = 0.0001). None of the other variables were correlated with CO changes after volume expansion (Table 4). The highest AUC values were found for ΔCO (0.78 ± 0.06; 95 % confidence interval (CI) [0.66-0.88], cIVC at baseline (0.62 ± 0.07; 95 %CI 0.49-0.74) and IVCmax at baseline (0.62 ± 0.07; 95 %CI 0.49-0.75) (Table 4, Fig. 1).

**Table 4:** Pearson correlation coefficient and area under the ROC curve for the various parameters

	Correlation coefficient (*p* value)	AUC ± SE
IVCmax at baseline	0.17 (0.21)	0.62 ± 0.07
cIVC at baseline	0.20 (0.12)	0.62 ± 0.07
ΔCO	0.69 (0.0001)	0.78 ± 0.06

*ROC* receiver operating characteristic, *AUC* area under the ROC curve ± standard error, *∆CO* change in cardiac output between baseline and after PLR, *IVCmax* maximum inferior vena cava diameter, *cIVC* inferior vena cava collapsibility index

**Fig. 2:**
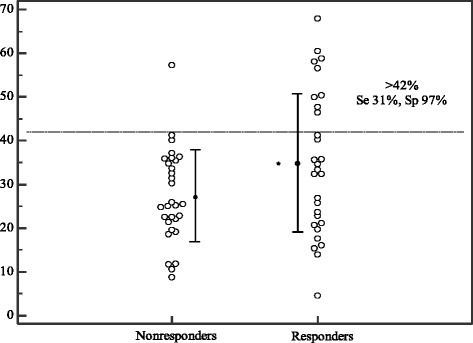
Inferior vena cava collapsibility index at baseline (expressed as a percentage) in responders and nonresponders. Individual values (*open circles*) and the mean ± SD per group (*filled circles* and *solid lines*). *Se* sensitivity, *Sp* specificity. * *p* <0.05 vs. nonresponders

**Fig. 3:**
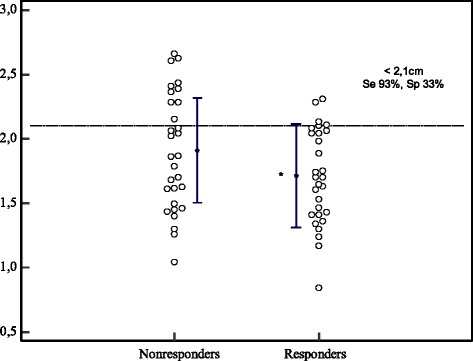
Maximum inferior vena cava diameter at baseline in responders and nonresponders. Individual values (*open circles*) and the mean ± SD per group (*filled circles* and *solid lines*). *Se* sensitivity, *Sp* specificity. * *p* <0.05 vs. nonresponders

In practical terms, a reduction in IVC diameter of 42 % or more in spontaneously breathing patients distinguished between responders and nonresponders with high specificity (97 %) and a positive predictive value (90 %) but low sensitivity (Fig. 2, Table 5). We found that IVCmax at baseline had little predictive value for fluid responsiveness (Table 5, Figs. 1 and 3). However, an increase in CO of 9.5 % or more during PLR distinguished responders from nonresponders with high specificity (87 %), a high positive predictive value (79 %), low sensitivity (52 %) and low negative predictive value (65 %) (Fig. 1).

**Table 5:** Accuracy of cIVC at baseline, IVCmax and ∆CO after PLR for predicting fluid responsiveness

	Se	Sp	LR+	LR-	PPV	NPV
cIVC > 42 %	31 %	97 %	9	0.7	90 %	59 %
IVCmax at baseline < 2.1 cm	93 %	33 %	1.4	0.2	57 %	83 %
ΔCO > 10 %	52 %	87 %	4	0.6	79 %	65 %

*∆CO* change in CO between baseline and after PLR, *cIVC* collapsibility index at baseline, *IVCmax* maximum diameter of the IVC, *PLR* passive leg raising, *Se* sensitivity, *Sp* specificity, *LR* likelihood ratio, *PPV* positive predictive value, *NPV* negative predictive value

## Discussion

Our results demonstrated that neither the IVC diameter nor IVC variability accurately predict fluid responsiveness in spontaneously breathing patients hospitalized in the ICU. Only inspiratory variations of IVC of 42 % or more may accurately predict an increase in CO after fluid infusion.

Echocardiography is a reliable guide to cardiac function in ICU patients - especially in cases of septic shock, in which there may be several overlapping causes of circulatory failure (e.g., hypovolemia, heart failure and vasoplegia) [16–18]. For many years, CVP was used to guide fluid infusion. The Surviving Sepsis Campaign guidelines still recommend assessing hypovolemia and fluid therapy by using CVP, which should be between 8 and 12 mmHg (2]. For a certain period of time, IVC diameter was used as a surrogate marker for right atrial pressure (RAP). Indeed, dilatation of the IVC was proven to be a reliable, sensitive marker of elevated CVP [19, 20]. The diameter of the IVC is easily recorded by transthoracic echocardiography in a subcostal view. The diameter is usually measured at end-expiration and end-diastole (with M-mode electrocardiographic (ECG) synchronization in a short-axis view, 2 cm below the right atrium) or at end-expiration (without ECG synchronization, using the two-dimensional long-axis view at the same location in the supine position) [10, 11, 21, 22]. These parameters were found to be well correlated with RAP in spontaneously breathing patients [23, 24]. Conflicting findings have been reported in mechanically ventilated patients: Benjelid et al. observed a good correlation [10], whereas Jue et al. [10] and Nagueh et al. [21] found a much weaker correlation. The IVC diameter at the cavo-atrial junction was also used to estimate CVP in a large cohort of mechanically ventilated patients [9], with a good correlation with RAP. From a practical point of view and considering these studies as a whole, a low IVC diameter (<10-12 mm) usually corresponds to low RAP (<10 mmHg) and seems to be an excellent indicator of fluid needs in patients in septic shock. In the present study, only one patient (a responder) had an IVC diameter below 10 mm.

In spontaneously breathing patients, decreased intrathoracic pressure and increased intra-abdominal pressure during inspiration increase the venous return [25]. The diameter of the IVC may then fall, due to decreased IVC transmural pressure (i.e., the intraluminal pressure less the extraluminal pressure). During insufflation (for the same intrathoracic pressure variation), higher RAP (and, therefore, a greater IVC pressure) will increase the IVC's transmural pressure, resulting in loss of IVC respiratory variability. Many researchers have reported a good correlation between RAP and IVC respiratory variability in spontaneously breathing patients [25–29]. In echocardiographic measurements, the cIVC (reflected by a caval index greater than or equal to 50 %) indicated a RAP value below 10 mmHg and caval indices below 50 % indicated a RAP value of 10 mmHg or more in the study by Kircher et al. [26]. Recently, Breenan et al. [29] reappraised the use of IVC variations to estimate the RAP; they analyzed IVCmax, IVCmin and cIVC during passive respiration and during a sniff test in which the patient was asked to perform a brief, rapid inspiration [29]. A cut-off value of 20 % for the passive cIVC and cut-off value 40 % in the sniff test were able to identify patients with RAP values less than or greater than 10 mmHg (AUC ROC: 0.93 and 0.91, respectively). In Breenan et al.'s study, a small or normal IVC diameter (<21 mm) and a sniff test result greater than 55 % were highly predictive of RAP < 10 mmHg.

In all these studies, RAP was used to assess fluid needs in patients with shock. Although an international working group [2] recently recommended the use of CVP as a marker of fluid responsiveness, this approach is highly controversial in ICU patients with shock [1, 8, 30]. In many studies, RAP and fluid responsiveness were either not correlated or only weakly correlation [1, 8]. Over the last decade, dynamic measurements (rather than static measurements) have become popular for predicting fluid responsiveness [1, 5–7, 12–16, 30–34]. Many different parameters have been found to be highly predictive of greater CO after fluid infusion in mechanically ventilated patients and spontaneously breathing patients. Vieillard Baron et al. demonstrated that the superior vena cava collapsibility index (recorded via transesophageal echocardiography) was very effective for assessing fluid needs in shocked and mechanically ventilated patients [31]. Using transthoracic echocardiography, Feissel et al. [12] and Barbier et al. [13] demonstrated that IVC variations were closely correlated with the CO increase after fluid infusion. Unfortunately, however, no information concerning the efficacy of assessing IVC in spontaneously breathing patients in terms of fluid responsiveness was reported in these publications. From a practical point of view, a cIVC of 42 % or more was a very accurate predictive marker of fluid responsiveness in spontaneously breathing patients. Neither IVCmax nor IVCmin were predictive of fluid responsiveness. These findings are similar to those recently published by Muller et al. (14) who found that cIVC > 40 % permitted the prediction of fluid responsiveness. In contrast, they found that cIVC < 40 % did not rule out fluid needs.

This study has a number of limitations. The study population was small and so a large-scale study must be conducted to confirm these findings. The CVP was not measured as a comparator for fluid responsiveness of patients, although this correlation has been widely confirmed elsewhere. Lastly, a sniff test was not performed.

## Conclusions

In conclusion, we analyzed the IVC diameter and its variability in spontaneously breathing patients with suspected hypovolemia. The IVCmax was not predictive of fluid responsiveness. In contrast, we found that cIVC > 42 % may predict an increase in CO after fluid infusion in spontaneously breathing patients in the ICU.

## Key messages

- In ICU spontaneously breathing patients with hypovolemia, respiratory variations of inferior vena cava > 42 % have a high specificity to predict an increase of cardiac output after fluid infusion

## Abbreviations

AA: Aortic area
AoD: Aortic diameter
AUC: Area under the curve, cIVC, Inferior vena cava collapsibility index
CO: Cardiac output
CVP: Central venous pressure
DBP: Diastolic blood pressure
HR: Heart rate
ICU: Intensive care unit
IVC: Inferior vena cava
IVCmax: Inferior vena cava maximum diameter
IVCmin: Inferior vena cava minimum diameter
PLR: Passive leg raising
PP: Pulse pressure
RAP: Right atrial pressure
ROC: Receiver operating characteristic
SAPS II: Simplified Acute Physiology Score II
SBP: Systolic blood pressure
SD: Standard deviation
SV: Stroke volume
VTI: Velocity-time integral
ΔCO: Change in SV between baseline and PLR
ΔSV: Change in SV between baseline and PLR
ΔVTI: Change in VTI between baseline and PLR
